# An acidic oligopeptide displayed on AAV2 improves axial muscle tropism after systemic delivery

**DOI:** 10.1186/1479-0556-10-3

**Published:** 2012-06-18

**Authors:** Ni-Chung Lee, Darin J Falk, Barry J Byrne, Thomas J Conlon, Nathalie Clement, Stacy Porvasnik, Marda L Jorgensen, Mark Potter, Kirsten E Erger, Rachael Watson, Steven C Ghivizzani, Hung-Chuan Chiu, Yin-Hsiu Chien, Wuh-Liang Hwu

**Affiliations:** 1 Departments of Pediatrics and Medical Genetics, National Taiwan University Hospital and National Taiwan University College of Medicine, Taipei, Taiwan; 2 Department of Pediatrics, Div. Cell and Molecular Therapy and Pediatric Cardiology, University of Florida, Gainesville, FL, USA; 3 Powell Gene Therapy Center, University of Florida, Gainesville, FL, USA; 4 Department of Pathology and Laboratory Medicine and Neuroscience, University of Florida, Gainesville, FL, USA; 5 Departments of Orthopaedics and Rehabilitation and Molecular Genetics & Microbiology, University of Florida, Gainesville, FL, USA; 6 Department of Pediatrics and Medical Genetics, National Taiwan University Hospital, 7 Chung-Shan South Road, Taipei, 100, Taiwan

**Keywords:** Adeno-associated virus, Tropism, L-aspartic acid, Acidic oligopeptide

## Abstract

**Background:**

The appropriate tropism of adeno-associated virus (AAV) vectors that are systemically injected is crucial for successful gene therapy when local injection is not practical. Acidic oligopeptides have been shown to enhance drug delivery to bones.

**Methods:**

In this study six-L aspartic acids (D6) were inserted into the AAV2 capsid protein sequence between amino acid residues 587 and 588. 129SVE mice were injected with double-stranded wild-type- (WT-) or D6-AAV2 mCherry expression vectors (3.24 x 10^10^ vg per animal) via the superficial temporal vein within 24 hours of birth.

**Results:**

Fluorescence microscopy and quantitative polymerase chain reaction confirmed higher levels of mCherry expression in the paraspinal and gluteus muscles in the D6-AAV2 injected mice. The results revealed that although D6-AAV2 was less efficient in the transduction of immortalized cells stronger mCherry signals were detected over the spine and pelvis by live imaging in the D6-AAV2-injected mice than were detected in the WT-AAV2-injected mice. In addition, D6-AAV2 lost the liver tropism observed for WT-AAV2.

**Conclusions:**

An acidic oligopeptide displayed on AAV2 improves axial muscle tropism and decreases liver tropism after systemic delivery. This modification should be useful in creating AAV vectors that are suitable for gene therapy for diseases involving the proximal muscles.

## Introduction

Adeno-associated virus serotype 2 (AAV2) is a non-pathogenic parvovirus that is commonly used in human gene therapy [1]. The advantages of AAV2 vectors include the ability to infect non-dividing cells, sustained gene expression, and low immune responses [2,3], but excessive liver tropism of these vectors and inefficient transduction of other organs after systemic injection is problematic. Other AAV serotypes have altered tissue tropism and higher infectivity than AAV2 [4,5]. However, specific targeting is still a problem; for example, the transduction of the liver can induce innate immune responses and toxicity [6,7]. Currently, studies focused on either genetic [8] or biochemical modifications [9] of the AAV capsid protein are ongoing.

Membrane-associated heparan sulfate proteoglycan (HSPG) is a receptor for the AAV2 virus [10]. The HSPG-binding motif is composed of the amino acid residues R484, R487, K532, R585, and R588 of the capsid protein (according to the numbering for VP1) [11]. The insertion of an oligopeptide between residues 587 and 588 disrupts the HSPG-binding motif [12] and has been shown to retarget AAV2 vectors to the angiogenic vasculature, diaphragm, heart, or endothelial cells [13-15].

The limited delivery of drugs to bones has been a barrier in the treatment of diseases of the skeletal system. Kasugai *et al.* demonstrated that proteins conjugated with an oligopeptide composed of six or more acidic amino acids (L-aspartate or L-glutamate) bound strongly to hydroxyapatite, a major component of bones [16]. Oligopeptide-conjugated enzymes were later developed to treat diseases including hypophosphatasia [17] and mucopolysaccharidosis type IV [18]. An AAV8 vector expressing deca-aspartate-tagged tissue-nonspecific alkaline phosphatase has also been used to treat hypophosphatasia [19].

In this study, we inserted six L-aspartic acids (D6) between residues 587 and 588 of the AAV2 capsid protein. We demonstrate that D6-AAV2 exhibits improved axial muscle tropism and decreased liver tropism after systemic delivery.

## Methods

### Plasmids and cell cultures

A nucleotide sequence (5'-GACGATGACGATGACGAT) encoding six L-aspartic acids (D6) was inserted between amino acid residues 587 and 588 of the AAV2 *cap* gene in the pAAV-RC plasmid (Agilent, Santa Clara, CA) using the QuikChange Lightning Site-Directed Mutagenesis Kit (Agilent). Double-stranded WT- and D6-AAV2 mCherry expression vectors were produced at the Powell Gene Therapy Center using methods previously reported [11]. HEK293 cells were harvested and purified using a discontinuous iodixanol gradient and concentrated in an Apollo® concentrator with a final volume of 500 μl. Viral genome (vg) titers were determined using quantitative real-time PCR (qPCR) with primers directed against the CMV promoter region [20]. The capsid proteins were analyzed by sodium dodecyl sulfate–polyacrylamide gel electrophoresis and silver staining. The heparan binding capability was measured by loading a known amount of WT- (4.95 × 10^11^ vg/ml) or D6-AAV2 (3.90 × 10^11^) into a heparin agarose column for chromatography [21]. The flowthrough and eluate were collected, and qPCR was performed to determine the viral genome titer. HT1080 cells, HEK293 cells, C2C12 cells, and human chondrocytes were seeded in 96-well plates and reached 80% confluence 24 hours later. Cells were infected with the WT- or D6-AAV2 vector at a multiplicity of infection (MOI) of 1,000 for HEK293 cells, 10,000 for C2C12 cells, and 20,000 for HT1080 cells and human chondrocytes. HEK293 and C2C12 cells were infected in the presence of a helper adenovirus according to the infectivity assay protocol for HEK293 cells [21]. Forty-eight hours after infection, the numbers of mCherry-positive cells were counted by fluorescence microscopy.

### Animal studies

Neonatal 129SVE mice were injected with the WT- or D6-AAV2 vector (n = 7 each group with 3.24 × 10^10^ vg per animal in 30μL) within 24 hours of birth via the superficial temporal vein using a 29 G needle. Live imaging was performed on anesthetized mice (n = 3 each group, monthly up to 4 months after injection, under 2% isoflurane in 100% O2) using the Xenogen IVIS® Spectrum *in vivo* Bioluminescence and Fluorescent Imager (Caliper Life Sciences, Hopkinton, MA), and mCherry signals were obtained with excitation/emission wavelengths of 570/620 nm. Mice were euthanized under isoflurane anesthesia prior to tissue collection via thoracotomy and removal of the heart. For direct fluorescence microscopy, mouse tissue was fixed in 4% paraformaldehyde after direct dissection of the tissue, embedded in OCT and cut into 6–12 μm sections (Leica CM1850 cryotome, Houston, TX, USA). A Chroma 51006 FITC/Texas Red dual-band filter was used to eliminate autofluorescence (Chroma, Bellows Falls, VT). The tissues were also stained with 4’,6-diamidino-2-phenylindole (DAPI). Tissue samples from another 4 mice in each group were also harvested for DNA extraction using the DNeasy Blood & Tissue kit (QIAGEN, Valencia, CA, USA) 4 weeks after injection. The total vg copy numbers in the tissues were determined by qPCR and expressed as vg per μg DNA. All animal studies were approved and performed in accordance with guidelines of the University of Florida Institutional Animal Care and Use Committee (IACUC No. 201004974) and the National Taiwan University College of Medicine and College of Public Health Institutional Animal Care and Use Committee (IACUC No. 20110265).

### Statistical analysis

Data are presented as the mean ± SD in each figure. Statistical analyses were performed using the SPSS (Statistical Package for the Social Sciences) statistical package, version 11.5. The Mann–Whitney Rank Test was used for comparisons of different groups. A *p* value less than 0.05 was considered statistically significant.

## Results and discussion

### Production of the D6-AAV2 vector and its infectivity in cells

**Figure 1:**
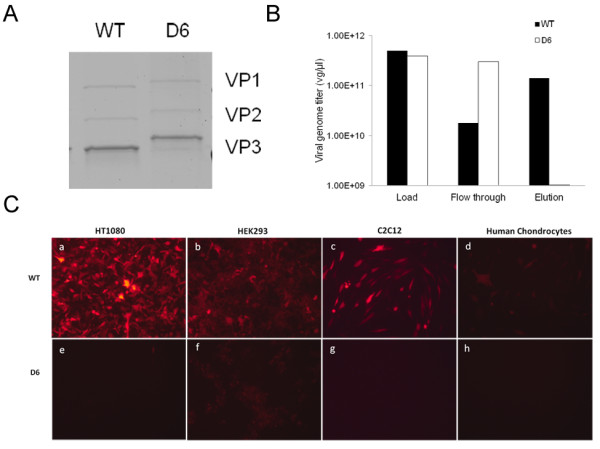
**Production of D6-AAV2 vectors and*in vitro*infection assay.** (**A**) Electrophoresis and silver staining for the WT- and D6-AAV2 capsid proteins. The picture shows capsid proteins VP1, VP2, and VP3. The three capsid proteins of D6-AAV2 were present at similar relative quantities but had higher molecular weights than those of WT-AAV2. (**B**) Heparin agarose chromatography. When known amounts of WT- and D6-AAV2 were loaded onto a heparin agarose column, the D6-AAV2 viruses did not bind to the column and were present in the flowthrough fraction. (**C**) Infectivity in cells shown by the mCherry red fluorescence. HT1080 cells (a and e), HEK293 cells (b and f), C2C12 cells (c and g), and human chondrocytes (d and h) were infected with either WT- or D6-AAV2. Compared with the WT-AAV2-infected cells, the D6-AAV2-infected cells showed either weak fluorescence (HEK293) or no fluorescence (all other types of cells).

D6-AAV2 was produced with a vg titer similar to that of WT-AAV2 (1.22x10^11^ and 1.08x10^11^ vg/ml, respectively, from 1 cell factory). Silver staining showed similar relative quantities of the VP1, VP2, and VP3 capsid proteins, but the capsid proteins from D6-AAV2 had higher molecular weights than those from WT-AAV2 (Figure 1A). D6-AAV2 lost its heparin-binding ability (Figure 1B), and therefore a heparin-agarose column could not be used for vector preparation. D6-AAV2 also lost the ability to infect cultured HT1080 cells, HEK293 cells, C2C12 cells, and human chondrocytes, which could all be infected by WT-AAV2 (Figure 1C).

### Tropism of D6-AAV2

**Figure 2:**
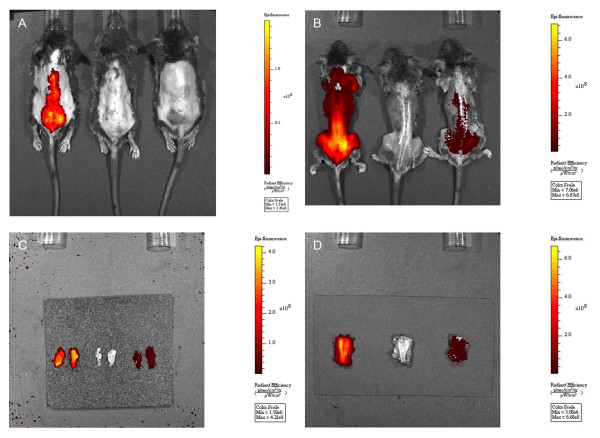
**IVIS live imaging for three mice at four months after injection.** The D6-AAV2-injected mouse is on the left, the lactated Ringer-injected mouse is in the middle, and the WT-AAV2-injected mice is on the right. (**A**) An image with the fur removed. (**B**) An image with the skin removed. (**C**) Dissected gluteus muscles. (**D**) Dissected paraspinal muscles. A scale bar was placed on each image. Stronger mCherry fluorescence was observed in the D6-AAV2-injected mouse over the spine and pelvis (A and B) and over the dissected gluteus (C) and paraspinal (D) muscles.

**Figure 3:**
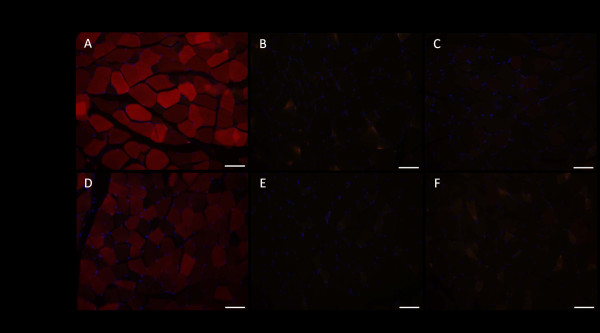
**Direct fluorescence microscopy for sections of the paraspinal and gluteus muscles from a D6-AAV2-injected mouse (A and D), a control mouse (B and E), and a WT-AAV2-injected mouse (C and F).** Red mCherry fluorescence, visualized using a FITC/Texas Red dual-band filter, was stronger in muscles from the D6-AAV2-injected mouse.

**Figure 4:**
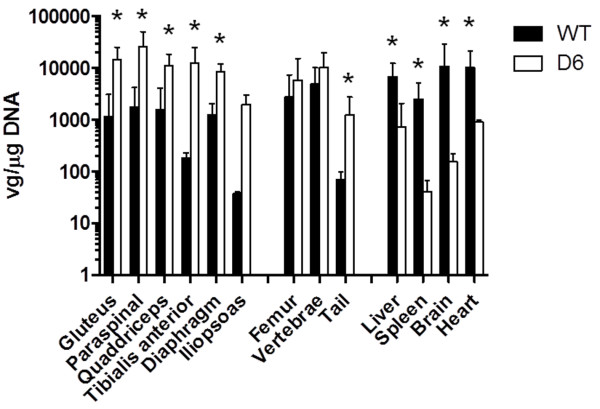
**Biodistribution of the WT- (n = 4) and D6-AAV2 (n = 4) vectors in mice four weeks after injection.** The vg copy numbers are shown on a log scale on the left. The error bars indicate one standard deviation. *P < 0.05.

IVIS live imaging was performed on mice monthly up to four months after injection. The results revealed that the mCherry signals were stronger over the spine and pelvis in the D6-AAV2-injected mice than in the WT-AAV2-injected mice (Figure 2A). Because 129SVE mice had dark fur, we needed to remove the fur before IVIS imaging. After we euthanized the mice and removed the skin, we repeated the IVIS imaging to confirm the mCherry signals (Figure 2B). We further demonstrated that the mCherry signals were from muscles and not bones by separating the gluteus (Figure 2C) and paraspinal muscles (Figure 2D) from the bones. By direct fluorescence microscopy, we could see strong mCherry red fluorescence in the paraspinal muscle sections in the D6-AAV2-injected mice (Figure 3A). Signals over the gluteus muscles were also visible (Figure 3D). However, there was no mCherry red fluorescence in either the WT-AAV2-injected mice or the control mice (Figure 3). qPCR quantification demonstrated much higher AAV2 copy numbers in the paraspinal and gluteus muscles and higher copy numbers in the diaphragm, quadriceps, tibialis anterior, and iliopsoas muscles in the D6-AAV2-injected mice than in the WT-AAV2-injected mice (Figure 4). In contrast, the copy numbers were lower in liver, spleen, heart, and brain in the D6-AAV2-injected mice (Figure 4). The study by Yu *et al*. demonstrated that the insertion of amino acids into the capsid of AAV2 at residue 587 can enhance this virus’s tropism for muscles, especially the lung, heart, and diaphragm, in adult mice [13]. Although D6-AAV2 also demonstrated higher tropism for muscle, the distributions of the muscle groups are different. This difference might due to the different effects of the insertions on tropism. In the study of Yu *et al*., the muscle-targeting peptide they inserted possessed neutral charges, whereas our D6 peptide had negative charges. The negative charges might lead to binding to hydroxyapatite in the bone. Additionally, the mice that we injected were in the neonatal period. The age of the mice might also have contributed to the different results.

### Proposed mechanisms for the altered tropism

The insertion of six L-aspartic acids into the capsid protein enhances AAV2 axial and hind limb muscle tropism and decreases liver tropism. It is known that the insertion of oligopeptides after amino acid residue 587 disrupts the HSPG-binding motif of AAV2 [12], leading to liver detargeting. However, acidic oligopeptides should bind to hydroxyapatite and lead to bone targeting [16]. It is possible that the binding between the acidic oligopeptides and hydroxyapatite in large bones including the vertebrae and pelvis only temporarily retains the virus. Because D6-AAV2 does not readily infect the bone marrow cellular components, the viruses ultimately leave the bones and infect the nearby muscles.

### Future applications

Many hereditary myopathies, including Duchenne muscular dystrophy, limb girdle muscular dystrophy, and adult-onset Pompe disease, preferentially involve the proximal and trunk muscles [22]. Therefore, the D6 modification would be helpful in designing viral vectors to treat those diseases. Because of the low yield of the AAV2 vector, we were not able to test D6-AAV2 in adult mice at the same dose per body weight as we used in the neonatal mice. There are already new AAV serotypes that have much higher transduction efficiencies than AAV2, such that addition of the D6 modification to those AAV vectors may further increase their relative muscle tropism and allow these vectors to be used in a safer manner in the treatment of hereditary myopathies.

## Conclusion

In this study, we demonstrated that an acidic oligopeptide displayed on AAV2 improves axial muscle tropism and decreases liver tropism after systemic delivery. This modification should be useful in creating AAV vectors suitable for gene therapy for diseases involving the proximal muscles. Future studies of the targeting effects of different acidic oligopeptides in conjunction with different AAV serotypes may be helpful to expand the applications of AAVs.

## Abbreviations

AAV, Adeno-associated virus; AAV2, Adeno-associated virus serotype 2; D6, Six L-aspartic acids; HSPG, Heparan sulfate proteoglycan; qPCR, Quantitative real-time PCR; MOI, Multiplicity of infection.

## Competing interests

The authors have no competing financial interests.

## Authors' contributions

Dr. NCL, Dr. DJF, and Dr. WLH designed the study, conducted the literature search, and drafted the manuscript. Dr. NC, Mr. MP, and Dr. HCC participated in the vector production, purification and titering. Ms. MLJ, Dr. RW, and Ms SP were involved in the animal care, tissue harvesting and pathology work. Dr. TJC and Ms. KEE were involved in the qPCR analysis of the viral distribution. Dr. BJB, Dr. SG, and Dr. YHC helped design the study design. All authors read and approved the final manuscript.
